# Decoding Immune Dynamics in Pregnant Women: Key Gene Expression Changes Following Influenza Vaccination

**DOI:** 10.3390/ijms26083765

**Published:** 2025-04-16

**Authors:** Rasha Elsayim, Manal M. Alkhulaifi, Abeer S. Aloufi, Razaz Abdulaziz Felemban, Lienda Bashier Eltayeb, Asawir Esamaldeen Ebrahim Mohamed, Hanan O. Alshammari, Esra’a Abudouleh

**Affiliations:** 1 Department of Botany and Microbiology, College of Science, King Saud University, Riyadh 11451, Saudi Arabia; relsayim.c@ksu.edu.sa (R.E.); manalk@ksu.edu.sa (M.M.A.); 2 Department of Biology, College of Science, Princess Nourah bint Abdulrahman University, P.O. Box 84428, Riyadh 11671, Saudi Arabia; asaloufi@pnu.edu.sa; 3 Department of Basic Medical Sciences, College of Medicine, King Saud Bin Abdulaziz University for Health Sciences, Jeddah 22384, Saudi Arabia; felembanr@ksau-hs.edu.sa; 4 King Abdullah International Medical Research Centre, Jeddah 22384, Saudi Arabia; 5 Department of Medical Laboratory Sciences, College of Applied Medical Sciences, Prince Sattam Bin Abdulaziz University, Al-Kharj 11942, Saudi Arabia; l.eltayeb@psau.edu.sa; 6 Department of Genetics and Animal Breeding, Faculty of Animal Production, University of Khartoum, Khartoum 13314, Sudan; asawir.esamaldeen@uofk.edu; 7 Department of Pharmaceutics, College of Pharmacy, Northern Border University, Rafha 76321, Saudi Arabia

**Keywords:** transcriptomic analysis, pregnant women, influenza, vaccine, hub genes, interferon pathway

## Abstract

Pregnant women are at an increased risk of severe influenza complications, necessitating vaccination as a preventive measure. Despite World Health Organization (WHO) recommendations for influenza vaccination during pregnancy, vaccination rates remain suboptimal in many regions. This study aims to identify key differentially expressed genes (DEGs) and biological pathways modulated by influenza vaccination in pregnant women pre- and post-vaccination, contributing to improved vaccine strategies. Microarray data from gene expression omnibus GEO dataset GSE166545 was analyzed to identify DEGs in blood samples from pregnant women at three time points: pre-vaccination (Day 0) and post-vaccination (Days 0 and 1) (Days 1 and 7). DEGs were filtered using an adjusted *p*-value < 0.05 and |log2 fold change| ≥ 1. Protein/protein interaction (PPI) networks, hub gene identification, and pathway enrichment analyses were conducted using STRING, Cytoscape, Kyoto Encyclopedia of Genes and Genomes (KEGG), and Reactome databases. Hub gene validation was performed using the Human Protein Atlas (HPA) and GTEx Portal. The GSE166545 dataset analysis revealed 60 up-regulated and 12,854 down-regulated genes (Day 1 vs. 7), 55 up-regulated and 12,933 down-regulated genes (Day 0 vs. 1), and two up-regulated with no down-regulated genes (Day 0 vs. 7). Key pathways included interferon alpha/beta (IFN-γ\ β) signaling and toll-like receptor signaling (TLR). Hub genes such as GBP1, CXCL10, RSAD2, and IFI44 demonstrated robust up-regulation, correlating with enhanced immune responses. The initial observation of JCHAIN’s notable up-regulation occurred on the seventh day following vaccination. Validation confirmed these genes’ roles in antiviral defense mechanisms and vaccine responses. The findings reveal distinct immune response dynamics in pregnant women following influenza vaccination, highlighting potential biomarkers for vaccine efficacy. This study underscores the importance of tailored vaccine strategies to improve maternal and neonatal outcomes.

## 1. Introduction

Pregnant women are at an elevated risk of severe influenza [1], necessitating vaccination during pregnancy to protect both the mother and her unborn or newborn child from infectious diseases [2]. Since 2005, the WHO has recommended influenza vaccination during the influenza season for all pregnant women. Specifically, those in the second trimester or later should receive an inactivated quadrivalent influenza vaccine [2]. This vaccination reduces influenza-associated morbidity and mortality during pregnancy and provides newborns with immune protection during their initial months [1]. Maternal immunization represents a safe and efficacious method of transferring maternal antibodies to neonates, ensuring passive immunity against potentially fatal infections until they can develop their own adaptive immune systems [2]. However, despite the existence of comprehensive national guidelines, maternal influenza vaccination rates remain suboptimal in numerous WHO European Region countries [1].

The immunological response to influenza vaccination encompasses a sophisticated interplay between innate and adaptive immune mechanisms. Innate immunity provides swift initial defense through neutrophils, macrophages, and dendritic cells (DCs), which serve as crucial antigen-presenting cells bridging innate and adaptive responses. Pattern-recognition receptors (PRRs), notably TLRs, identify viral components such as influenza’s single-stranded RNA, initiating antiviral cascades. Surface TLRs detect microbial membrane elements, while intracellular TLRs (TLR3, TLR7, TLR9) recognize viral nucleic acids, activating pathways like MyD88-independent TRIF to orchestrate antiviral immunity. These processes induce cytokines, including type-1 interferons (IFNs), which restrict viral proliferation, promote DC maturation, and facilitate immune cell recruitment. Plasmacytoid dendritic cells (pDCs) produce substantial quantities of type-1 IFNs upon viral RNA/DNA detection, while natural killer (NK) cells contribute cytotoxic responses to manage viral burden. Adaptive immunity comprises homotypic immunity via neutralizing antibodies targeting specific influenza strains and heterotypic immunity mediated by CD4+ and CD8+ T cells, which generate cytokines such as IL-2 and IFN-γ for viral elimination and memory formation. During pregnancy, there is a significant modulation in immunity, characterized by altered TLR expression and cytokine profiles, including diminished type-1 IFN production, potentially affecting antiviral responses and elevating susceptibility to complications. The influenza vaccine stimulates innate immunity through TLRs and cytokines while bolstering adaptive immunity via specific antibodies and memory T cells, conferring both immediate and enduring protection, underscoring the intricate immune adaptations during gestation [3].

Gene expression studies offer a robust methodology for deciphering the intricacies of immune responses to influenza vaccination. Through the examination of transcriptomic data, scientists can elucidate pathways activated during immune responses, including those mediated by toll-like receptors (TLRs) and interferon-stimulated genes (ISGs) [4]. This approach enables the identification of pivotal hub genes that function as central regulators of immune activity, encompassing genes involved in cytokine signaling and T-cell activation. For instance, IFI27 and STAT1 have been associated with robust immune responses to vaccination [5]. These discoveries contribute to the development of biomarkers for assessing vaccine-induced immunity and predicting efficacy. Hub genes serve as potential indicators of vaccine efficacy and safety owing to their crucial roles in immune regulation. Their identification through gene co-expression network analysis allows researchers to differentiate between vaccine responders and non-responders [6].

The immunological alterations induced by pregnancy, which promote a more tolerant state to support fetal development, present challenges in comprehending the molecular-level modulation of immune responses following influenza vaccination. Recent studies indicate that pregnant women exhibit distinct immune responses compared to non-pregnant individuals, attributed to modifications in cytokine and TLR expression [7,8]. However, there remains a paucity of high-resolution data elucidating the impact of vaccination on specific molecular pathways, particularly those mediating innate and adaptive immune interactions. While advanced methodologies such as single-cell RNA sequencing offer the potential for deeper insights, their implementation in pregnant cohorts is currently limited. Addressing this research gap is crucial for the development of vaccines tailored to pregnancy-specific immunological milieus. In the interim, efforts should focus on identifying differentially expressed hub genes in pre- and post-vaccination blood samples from pregnant women, as well as delineating the biological pathways associated with these genes and their functions in immune response mechanisms.

The objective of this investigation is to identify hub genes that demonstrate differential expression in blood samples obtained from pregnant women before and after vaccination. Furthermore, this research aims to clarify the biological pathways associated with these genes and their involvement in immune response mechanisms. This study’s findings are expected to advance personalized medicine and vaccine strategies tailored for pregnant populations. Additionally, the research has the potential to inform targeted interventions that may improve maternal and neonatal health outcomes.

## 2. Results

This study employed bioinformatics analysis and experimental verification methods to explain potential biomarkers in pregnant women vaccinated with influenza vaccine (a quadrivalent inactivated influenza vaccine (QIV)) and to identify correlates of antibody responses from transcriptomics. We identified the primary up-regulated and down-regulated genes at Day 0 pre-vaccination, Day 1, and Day 7 post-vaccination and subsequently determined the main biological processes and pathways involved in the influenza vaccine response. These findings were then verified through the GTEx Portal (https://gtexportal.org/home (accessed on 15 October 2024)) and the HPA dataset.

**Figure 1:**
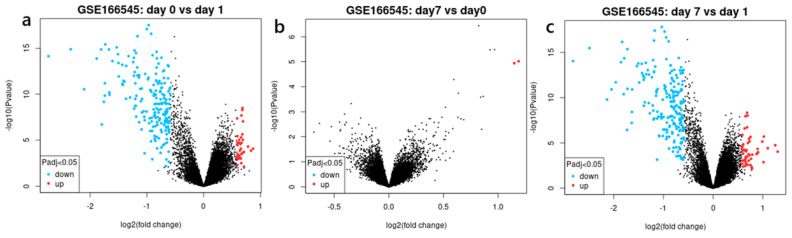
Volcano plots depicting the distribution of all differentially expressed genes (DEGs) in (**a**) GSE166545: Day 0 versus Day 1, (**b**) GSE166545: Day 7 versus Day 0, and (**c**) GSE166545: Day 7 versus Day 1. Red, blue, and black colors represent up-regulated genes, down-regulated genes, and genes with no significant difference in expression, respectively.

Differentially expressed genes are referred to as DEGs. These are genes whose expression levels exhibit statistically significant differences between two or more conditions or groups, such as treated versus untreated samples, diseased versus healthy tissues, or pre-vaccination versus post-vaccination states. The criteria to identify DEGs, *p* < 0.05, which ensures the changes are statistically significant, and |logFC| > 1.0, indicating a 4-fold increase in expression, yielded a total of 60 up-regulated genes and 12,854 down-regulated genes at Day 1 and 7 for the GSE166545 dataset. Additionally, a total of 55 up-regulated genes and 12,933 down-regulated genes were observed on Days 0 and 1 for the same dataset. Furthermore, on Days 0 and 7, two genes were up-regulated, and no down-regulated genes were observed (Figure 1a–c).

### 2.1. KEGG and Reactome Enrichment Analyses of DEGs

An enricher was employed to conduct Reactome functional analysis and KEGG pathway enrichment analysis of differentially expressed genes (DEGs) across various conditions. The results elucidate critical biological pathways and processes involved in immune activation and modulation post-vaccination in pregnant women.

### 2.2. Reactome Pathways

**Figure 2:**
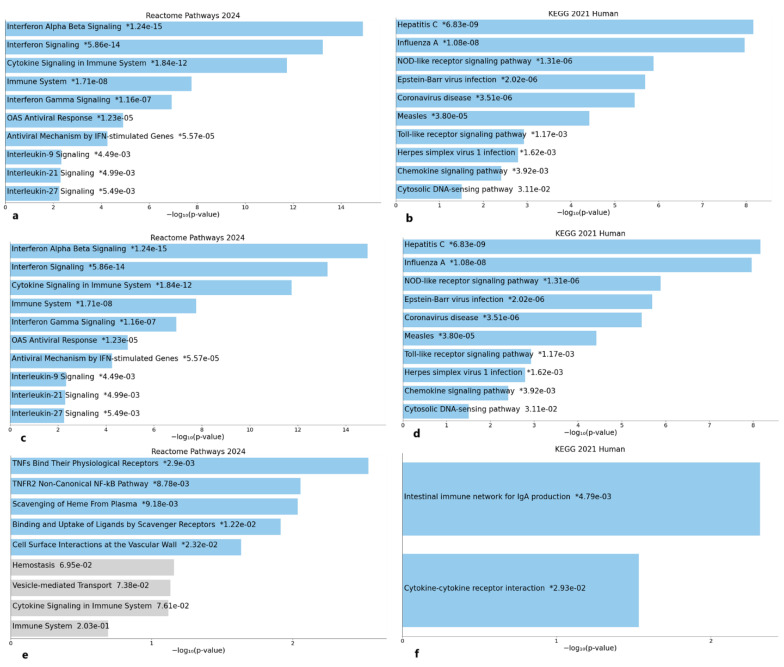
Significant Reactome pathways at Days 0–1, Days 1–7, and Days 0–7 (**a**,**c**,**e**), respectively. KEGG pathways at Days 0–1, Days 01–7, and Days 0–7 (**b**,**d**,**f**), respectively, enriched with DEGs. *—*p* value for each pathway.

A total of 15 enriched Reactome pathways were identified based on the analysis. Among these, the top pathways were significantly enriched and primarily associated with immune system responses. The most notable Reactome pathways include the following. 1. Interferon Alpha/Beta Signaling: This pathway was significantly enriched across conditions, indicating its crucial role in antiviral responses and the activation of innate immunity. 2. Cytokine Signaling in the Immune System: This pathway underscores the role of cytokine-mediated communication in coordinating immune responses. 3. Antiviral Mechanisms by IFN-Stimulated Genes: This pathway reflects the activation of downstream effectors critical for pathogen defense. 4. TNFs Bind Their Physiological Receptors: A late-stage pathway was observed, implicating the involvement of tumor necrosis factor-mediated signaling in immune regulation (Figure 2a,c,e).

### 2.3. KEGG Pathways

Concurrently, KEGG pathway enrichment analysis yielded 12 significant pathways. These pathways predominantly corresponded to key immune and infection-related processes, including the following. 1. Toll-like Receptor Signaling Pathway: A critical pathway for innate immune activation upon recognizing pathogen-associated molecular patterns (PAMPs). 2. NOD-like Receptor Signaling Pathway: This pathway implicates the involvement of intracellular pattern recognition and downstream inflammatory cascades. 3. Cytokine/Cytokine Receptor Interaction: A central pathway in orchestrating immune communication and activation. 4. Influenza A: This pathway aligns with vaccination dynamics and the immune response specific to viral challenges (Figure 2b,d,f).

**Figure 3:**
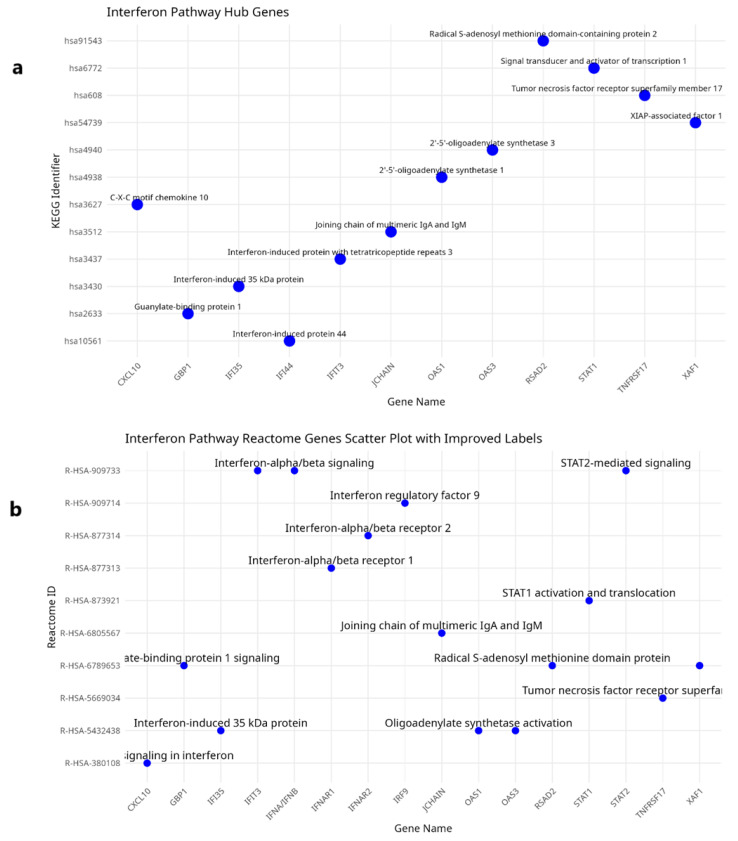
(**a**) Hub genes in the interferon alpha and beta KEGG pathway. (**b**) Hub genes in the interferon alpha and beta Reactome pathway.

The scatter plots presented in Figure 3a,b illustrate the hub genes associated with the interferon alpha and beta signaling pathway within the KEGG and Reactome datasets, respectively. These visual representations facilitate a comparative examination of the expression patterns and functional roles of the hub genes across both datasets. In the KEGG dataset, prominent hub genes such as STAT1, RSAD2, IFI35, and CXCL10 exhibit robust associations with the interferon alpha/beta signaling pathway. The distribution observed in this scatter plot suggests an elevated enrichment of genes implicated in direct immune responses, with a particular emphasis on antiviral mechanisms. Analogously, the Reactome dataset reveals significant mapping of hub genes, including STAT1, IFIT3, OAS1, and GBP1, within the interferon signaling pathway. Moreover, the data from Reactome appear to accentuate downstream signaling events, notably the transcriptional activation of interferon-stimulated genes (ISGs).

### 2.4. Observations Across Conditions

Day 0 to Day 1: Reactome pathways such as interferon signaling and OAS antiviral response were strongly enriched, highlighting the rapid innate immune activation following vaccination. Concurrently, KEGG pathways like toll-like receptor signaling demonstrated the immediate recognition and immune activation cascade (Figure 2a,b). Day 1 to Day 7: Reactome pathways transitioned to adaptive immune processes, with interleukin-21 and interleukin-27 signaling enriched. KEGG pathways indicated resolution and adaptive immunity via chemokine signaling (Figure 2c,d). Day 0 to Day 7: Sustained modulation was reflected in Reactome pathways such as TNFs Bind Their Physiological receptors, signifying immune regulation. KEGG pathways, including intestinal immune network for IgA production, highlighted the role of mucosal immunity and antibody response (Figure 2e,f).

**Figure 4:**
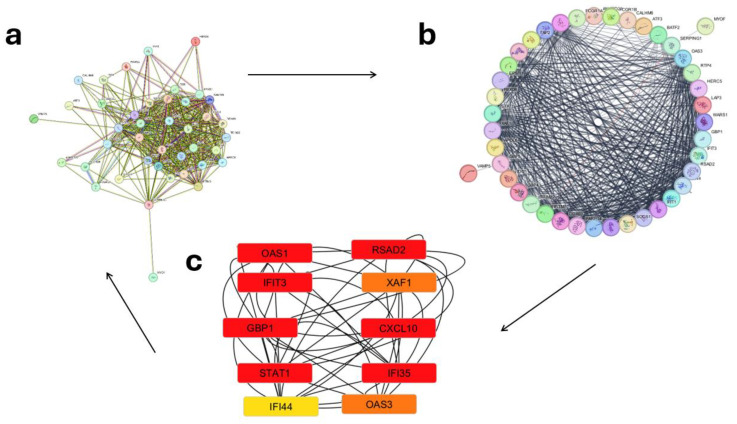
(**a**) Analysis of protein/protein interactions and identification of hub genes for pre- and post-vaccination periods (Days 0–1). The STRING database was used to predict interactions among the 56 up-regulated and 12,958 down-regulated differentially expressed genes (DEGs) common to both microarray datasets. (**b**) Data analysis was conducted using the Network Analyzer plugin for Cytoscape. (**c**) Subsequently, the Cytohubba plugin was employed to analyze hub genes and determine the top-ranking genes (top 10 presented). Red = High up-regulation of gene expression, Orange = Moderate up-regulation, and Yellow = Low up-regulation or mild expression change.

**Figure 5:**
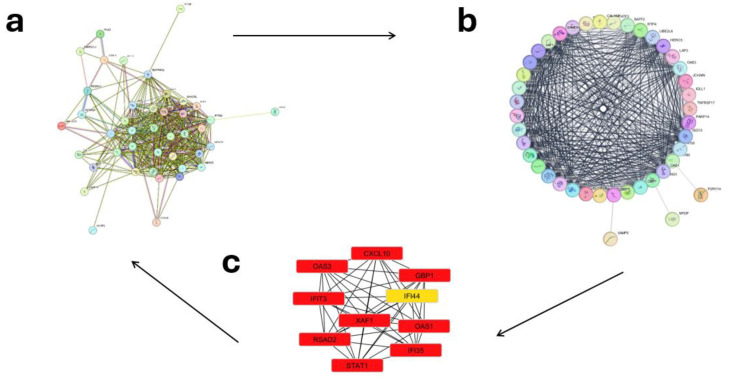
(**a**) Analysis of protein/protein interactions and identification of hub genes for post-vaccination periods (Days 1–7). The STRING database was used to predict interactions among the 60 up-regulated and 13,092 down-regulated differentially expressed genes (DEGs) common to both microarray datasets. (**b**) Data analysis was conducted using the Network Analyzer plugin for Cytoscape. (**c**) Subsequently, the Cytohubba plugin was employed to analyze hub genes and determine the top-ranking genes (top 10 presented). Red = High up-regulation of gene expression, Orange = Moderate up-regulation, and Yellow = Low up-regulation or mild expression change.

**Figure 6:**
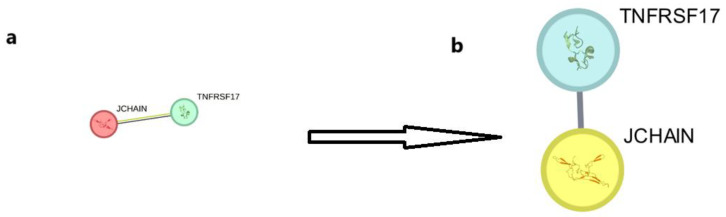
(**a**) Analysis of protein/protein interactions and identification of hub genes for post-vaccination periods (Days 0–7). The STRING database was used to predict interactions among the 2 up-regulated and 13,158 down-regulated differentially expressed genes (DEGs) common to both microarray datasets. (**b**) Represents both the Network Analyzer plugin for Cytoscap and the Cytohubba plugin to analyze hub genes and determine the top-ranking genes (top 2 presented).

The PPIs were examined for 60 up-regulated and 12,854 down-regulated genes (Day 1 vs. 7), 55 up-regulated and 12,933 down-regulated genes (Day 0 vs. 1), and 5 up-regulated with 12,847 down-regulated genes (Day 0 vs. 7). In order to further explore the biological roles of the identified DEGs, the STRING database was used to construct a PPI network. Then, we analyzed the network using Cytoscape and its tools, Network Analyzer and the cytoHubba plugin; the results are presented in Figure 4, Figure 5 and Figure 6. The 12 genes with the highest scores were defined as hub genes. On Days 0–1, Days 1–7, and 0–7, the genes IFI35, STAT1, RSAD2, OAS3, GBP1, IFI44, OAS1, XAF1, CXCL10, IFIT3, TNFRSF17, and JCHAIN are up-regulated with significant LogFc and *p*-value (Table 1). An examination of Table 1 reveals a hierarchical pattern of gene up-regulation in response to vaccination. Genes exhibiting the most pronounced up-regulation (LogFc ≥ 1.5) include GBP1, CXCL10, RSAD2, and IFI44, suggesting their critical involvement in the immune response. A second tier of genes, comprising STAT1, IFI35, OAS1, OAS3, IFIT3, XAF1, and TNFRSF17, demonstrates moderate up-regulation (1.0 ≤ LogFc < 1.5), indicating a significant yet comparatively reduced level of activation. JCHAIN, with its low up-regulation (LogFc < 1.0), appears to play a less prominent role in the immune cascade, as evidenced by its weaker statistical significance. The integration of LogFc values and *p*-values in this analysis underscores the likely primacy of GBP1, CXCL10, RSAD2, and IFI44 in orchestrating the vaccine-induced immune response.

### 2.5. Verification of Potential Biomarker Expression by Using GTEx and Human Atlas Protein Datasets

The hub genes were verified through bioinformatic analysis using two datasets, namely GTEx and HPA, to confirm their role in the immune system and vaccine response, as well as their associated pathways in the immune system. This verification process was conducted to corroborate our findings from GEO and Enrichr analyses, which incorporated KEGG and Reactome datasets, as well as STRING and Cytoscape. Furthermore, GTEx was utilized to detect gene expression at the transcriptomic level (RNA), while the HPA focused on protein-level validation in plasma. Consequently, the Human Atlas detected only six out of the twelve hub genes, namely, TNFRSF17, STAT1, OAS3, JCHAIN, IFI35, and GBP1. The remaining hub genes were not identified. Conversely, GTEx detected all hub genes, including JCHAIN, TNFRSF17, CXCL10, IFI44, GBP1, OAS3, STAT1, XAF1, OAS1, IFI35, RSAD2, and IFIT3.

**Figure 7:**
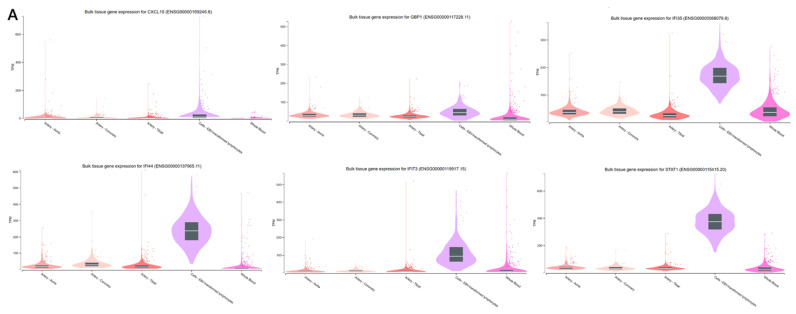

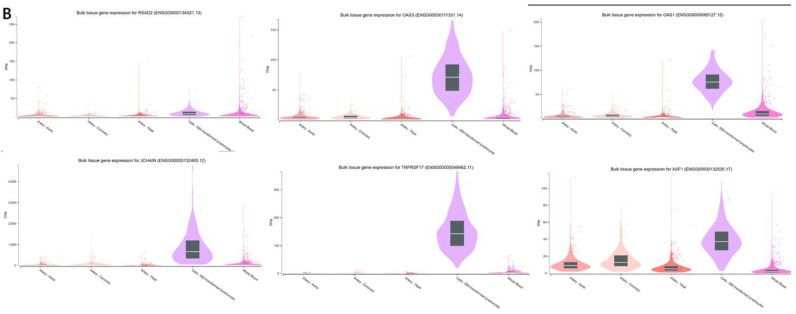
(**A**,**B**) GTEx dataset detected expression of all ten hub genes on Days (0–1) and (1–7) pre- and post-vaccination, respectively, and the two novel genes on Days (0–7) post-vaccination on the immune cells and their roles as antiviral and influenza vaccine pathways.

Analysis of Figure 7A,B reveals that all hub genes identified in the GTEx dataset are integral components of the immune system, contributing to antiviral defense mechanisms with varying degrees of involvement. Among these, JCHAIN and TNFRSF17 exhibited the highest expression levels in blood samples. JCHAIN demonstrated pronounced activity in EBV-transformed lymphocytes, highlighting its essential function in immunoglobulin production and immune cell modulation. The minimal presence of JCHAIN in arterial tissues further substantiates its specialized role in immune processes. Similarly, TNFRSF17 showed robust expression in immune cells, particularly EBV-transformed lymphocytes, while remaining virtually undetectable in systemic or vascular tissues, indicating its specific involvement in immune-related activities. This study marks the first instance of detecting both JCHAIN and TNFRSF17 on Days 0 and 7 following vaccination, elucidating their participation in influenza vaccine-related pathways. The pronounced expression of these genes in immune cells provides additional evidence for their specialized immunological functions. The gene CXCL10 demonstrates pronounced expression in lymphocytes and moderate levels in whole blood, underscoring its relevance in immune signaling with systemic implications. A cluster of genes, including IFI44, GBP1, OAS3, STAT1, XAF1, and OAS1, exhibit robust expression in lymphocytes, moderate presence in whole blood, and minimal activity in arterial tissues. This pattern collectively highlights their critical functions in antiviral responses and immune system regulation. Similarly, IFI35 and RSAD2 show comparable expression profiles, with high activity in lymphocytes and moderate expression in whole blood, indicative of their involvement in immune processes. Lastly, IFIT3 displays substantial lymphocyte expression and moderate systemic activity, reflecting its important role in immune signaling pathways.

**Figure 8:**
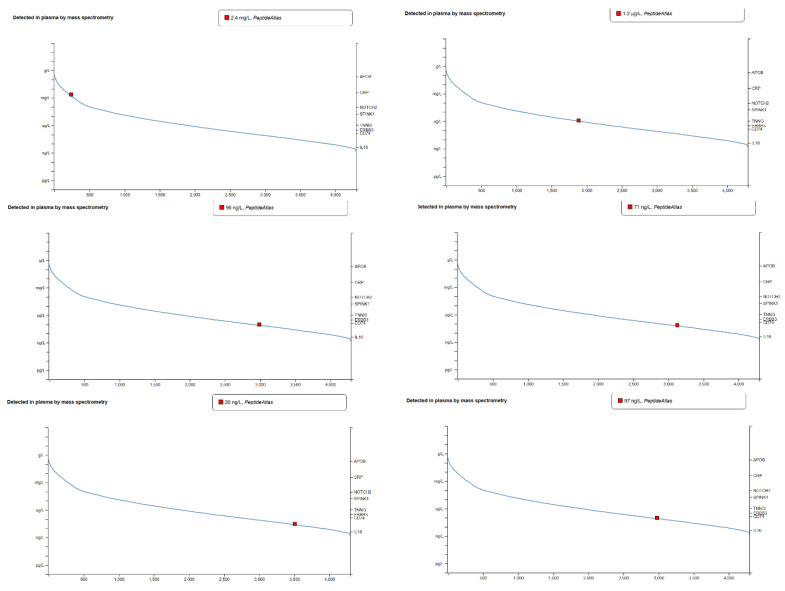
The Human Protein Atlas dataset detected expression of all ten hub genes on Days (0–1) and Days (1–7) pre- and post-vaccination, respectively, and the two novel genes on Days (0–7) post-vaccination on the immune cells and their roles as antiviral and influenza vaccine pathways.

Following the validation of our results using GETx, we subsequently validated the same genes within the HPA. The findings revealed unexpected results that corroborated our detection of novel genes. The analysis indicated that the high-abundance protein is JCHAIN, detected at 2.4 mg/L, establishing it as highly abundant and integral to humoral immunity. The moderate-abundance protein is TNFRSF17, present at 1.2 μg/L, correlating with its B-cell activity. The low-abundance proteins are STAT1, OAS3, IFI35, and GBP1, detected at ng/L levels, representing their regulatory roles and intracellular immune functions (Figure 8). This validation substantiates that the identified hub genes are integral to immune processes, with functions encompassing signaling, antiviral defense, and humoral immunity, thereby providing insights into their potential as targets for vaccines and therapeutic interventions against influenza.

**Figure 9:**
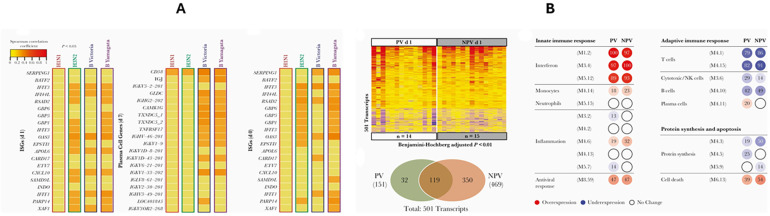
(**A**,**B**) Transcriptional profiles and modular expression analysis following influenza vaccination. (**A**) Heatmap showing differentially expressed genes (adjusted *p* < 0.05) across study participants, highlighting immune-related gene expression patterns. (**B**) Modular expression analysis at Day 1 post-vaccination comparing previously vaccinated (PV) and non-previously vaccinated (NPV) individuals. Overexpression (red), underexpression (blue), and unchanged expression (white) are indicated. Data cited from GSE166545.

Validation results indicated that JCHAIN and TNFRSF17 exhibited the highest expression levels in blood samples. To confirm the roles of these genes, detected on Days 0 and 7 post-vaccination, in the host immune response to the influenza vaccine, a correlation study was conducted between these genes and antibody titers using data from the GSE166545 dataset. It was found that TNFRSF17 correlates with increased antibody titers at Day 30 post-vaccination. Although JCHAIN is up-regulated, it does not show a direct correlation with antibody responses (Figure 9A). Furthermore, Figure 9B illustrates that transcriptional profiling and modular analysis on Day 1 post-vaccination revealed significant immune response differences between previously vaccinated (PV) and non-previously vaccinated (NPV) individuals. Notably, the plasma cell module (M4.11) was overexpressed in the PV group, consistent with findings on TNFRSF17, which plays a crucial role in B-cell activation and antibody responses. While JCHAIN was highly expressed in immune cells, its role did not directly correlate with antibody production, aligning with the observed modular expression patterns. These results further substantiate the specialized immunological functions of TNFRSF17 and JCHAIN in vaccine-induced immune responses.

## 3. Discussion

Vaccination during pregnancy can provide protection for both the expectant mother and the developing fetus and neonate against infectious diseases [9,10,11]. This study employs bioinformatics methodologies to investigate the biomarkers and hub genes in the signaling pathway of influenza vaccine pre- and post-vaccination during pregnancy. We analyzed the microarray dataset (GEO) to identify differentially expressed genes (DEGs) and hub genes associated with the influenza vaccine. A total of 12,914 shared DEGs (60 up-regulated and 12,854 down-regulated genes) were identified on Days 1–7 post-vaccination, 12,988 shared DEGs (55 up-regulated and 12,933 down-regulated genes) were detected on Days 0 pre-vaccination and 1 post-vaccination, and 12,849 shared DEGs (2 genes were up-regulated and 12,847 genes were down-regulated) in the GEO dataset. Subsequently, we elucidated their functions and pathways through Reactome and KEGG pathway enrichment analyses performed with the spring. The protein/protein interaction (PPI) network was visualized in Cytoscape, and the significantly dense modules were detected by the Maximal Clique Centrality (MCC). Genes exhibiting higher scores in the MCC analysis were considered hub genes, which are identical for Days 0–1 and 1–7 pre- and post-vaccination, respectively, while only two hub genes were identified for Days 0–7.

The analysis of differentially expressed genes (DEGs) reveals significant alterations in gene expression, indicative of active immune regulation. Up-regulated genes may be linked to processes such as antigen presentation or antibody production, while down-regulated genes potentially reflect the suppression of unnecessary inflammatory responses [4]. A systems biology approach was utilized to examine transcriptomic responses to influenza vaccination, and rapid activation of interferon-related genes and up-regulation of immune pathways within 24 h post-vaccination was observed. In contrast to the current study, which noted substantial gene down-regulation on Day 7, Nakaya and others’ research found that adaptive immune pathways remained active for up to one week [5]. This discrepancy may be attributed to differences in study populations or vaccine formulations. Another study by Sobolev and others conducted a transcriptomic analysis in young adults following influenza vaccination, identifying a peak in innate immune activation on Day 1 and subsequent engagement of adaptive immunity by Day 7 [12]. While this aligns with the gene up-regulation observed on Day 1 in the present study, it contrasts with the significant down-regulation noted on Day 7, suggesting that pregnancy may uniquely modulate immune activity [12]. The pathway analysis of GBP1 revealed its role in balancing immune activation and inflammation by amplifying interferon responses and interacting with p62 to enhance pathogen clearance. This aligns with Kim and others who found that GBP1 plays a critical role in regulating inflammation during viral infections, although their study focused on non-pregnant populations [13]. STAT1’s dual role in promoting antiviral responses and inflammatory cytokine production, particularly through Y701 phosphorylation, underscores its central position in both innate and adaptive immunity. However, Liu and his research group noted that excessive STAT1 activation can lead to hyper-inflammation, emphasizing the importance of tight regulation. This discrepancy in findings may be attributed to pregnancy’s unique immune modulation [14]. The disruption of lipid raft-associated processes crucial for viral replication is attributed to RSAD2, a finding that aligns with the research of Helbig and others on influenza virus replication inhibition [15]. Similarly, the OAS1-3 family impedes viral replication through RNase L activation and subsequent RNA degradation, as elucidated by Pabst and Slack, although their study did not specifically address pregnancy-related responses [16]. TNFRSF17’s involvement in T-cell expansion and cytokine response regulation underscores its significance in adaptive immunity and enduring protection. This is corroborated by Bartok and Hartmann [13], who reported enhanced memory T-cell responses post-vaccination due to TNFRSF17 [17]. The recruitment of immune cells by CXCL10 contributes to robust cellular immunity, as demonstrated by Raymond and others, who also noted potential risks of excessive inflammation, a concern mitigated during pregnancy through immune modulation [18]. According to our research, IFIT3 and IFI44, which are both essential for type I interferon signaling, have antiviral qualities through their ability to bind to and target viral RNA for destruction and suppression, respectively. Schneider and others observed comparable roles for these genes but cautioned that their pathways might differ across vaccine types and populations [19]. Interestingly, the JCHAIN gene was found to be up-regulated on Day 7. Based on its pathway, we discovered that it plays a role in polymerizing IgA and IgM, which suggests that this study may be novel because its up-regulation during influenza vaccination and pregnancy has not been documented before. JCHAIN encodes the joining chain of polymeric immunoglobulins (IgA and IgM), which play an essential role in mucosal immunity. Its increased expression on Day 7 post-vaccination indicates an active role in improving maternal humoral immunity, which might promote the transfer of protective antibodies to the fetus. When comparing our JCHAIN gene results to a previous study conducted on pregnant women following pertussis vaccination (Tregoning et al. 2020 [20]), we found no evidence of JCHAIN expression in their gene expression data. This suggests that vaccines stimulating mucosal immunity may have distinct effects on maternal and neonatal immune protection, highlighting the need for further investigation. This is consistent with JCHAIN’s established function in mediating immunoglobulin secretion across epithelial barriers, which is critical for neonatal passive immunity. The observed increase in JCHAIN expression could indicate a late-phase immune response, resulting in sustained antibody production and improved maternal and fetal influenza protection. However, further study is required to verify this finding and determine its clinical relevance in pregnancy-specific vaccine responses. Nevertheless, Pabst and others emphasized the importance of IgA transport in conferring passive immunity during pregnancy [16]. Many others observed attenuated CXCL10 expression in pregnant women following vaccination compared to non-pregnant women, suggesting a potential dampening of chemokine-driven immune responses during pregnancy [20,21,22].

The investigation of hub genes during the pre- and post-vaccination periods (Days 0–1 and Days 1–7, respectively) identified a set of 10 genes with the highest MCC scores: OAS1, RSAD2, IFIT3, XAF1, GBP1, CXCL10, STAT1, IFI35, IFI44, and OAS3. These genes demonstrate significant interconnectedness, indicating their crucial roles in the underlying biological mechanisms. Many of these genes are recognized as interferon-stimulated genes (ISGs), which are essential for immune and antiviral responses [23]. The study by Cao and others on children vaccinated with different influenza formulations reported that trivalent inactivated influenza vaccine (TIV) induced early expression of interferon (IFN)-related genes, correlating with robust antibody responses. Our analysis corroborates this observation, identifying interferon alpha/beta signaling as a significantly enriched Reactome pathway. Additionally, hub genes such as IFI35, IFI44, and OAS3 showed increased expression, reinforcing the critical role of IFN-mediated responses in vaccination outcomes. Unlike Cao et al., who examined pediatric responses, our study extends these findings to pregnant women, suggesting that IFN responses are pivotal across different populations [10].

In the immediate post-vaccination period, JCHAIN emerges as a prominent player in the network, directly influencing functional antibody production. TNFRSF17, while significant, plays a supportive role by regulating upstream B-cell activity, thereby facilitating JCHAIN’s critical function. This network exemplifies the intricate collaboration between genes involved in humoral immunity, which is fundamental to the body’s post-vaccination defense mechanisms. Genes exhibiting both high LogFC and high MC values are likely to be of the greatest biological significance, as they are not only strongly regulated but also central to the network. Examples such as OAS1 and RSAD2 may display these characteristics due to their vital roles in interferon-mediated immune responses. Genes exhibiting low LogFC but high MCC (STAT, IFI3, IFIT, OAS, XAF, and TNFRSF17) maintain their network significance despite minimal expression alterations. These genes potentially act as critical scaffolds or regulatory elements for pathways and interactions, thus playing pivotal roles in cellular or immune regulation irrespective of their stable expression levels. Conversely, genes characterized by high LogFC but low MCC (GBP, CXCL1, RSAD, IFI44, and OAS3) display marked expression changes yet may not be central to the network architecture [24]. Such genes might fulfill specialized or peripheral functions rather than operating within the core regulatory framework. Genes with both low LogFC and low MCC (JCHAIN) are likely less influential in terms of expression and network interactions within this particular context. Nevertheless, JCHAIN, despite its low LogFC and potential low MCC, contributes significantly to enhancing post-vaccination maternal humoral immunity, facilitating antibody-mediated passive immunity transfer to the fetus, and supporting the vaccine’s overarching aim of safeguarding both mother and fetus [22].

The validation of hub genes utilizing GTEx and HPA datasets offers compelling evidence supporting their involvement in immune responses, vaccine mechanisms, and related pathways. This comprehensive analysis corroborates findings from previous bioinformatic investigations (GEO, Enrichr, KEGG, Reactome, STRING, and Cytoscape), emphasizing their functional significance at both transcriptomic and proteomic levels. The GTEx dataset successfully identified all 12 hub genes, confirming their transcriptional activity in relevant tissues, particularly immune cells. The HPA dataset detected 6 hub genes (JCHAIN, TNFRSF17, STAT1, OAS3, IFI35, and GBP1) at the protein level, underscoring their contributions to immune and vaccine pathways. Both datasets validated STAT1, CXCL10, and OAS3, reinforcing their pivotal roles in immune processes and vaccination responses.

GTEx revealed expression patterns for the remaining genes not detected in plasma samples. The discrepancy between the GTEx and HPA datasets can be attributed to several factors. GTEx captures transcript levels across tissues but does not account for post-transcriptional modifications or protein localization [25]. Conversely, HPA only detects translated, stable proteins present in plasma. Genes with intracellular activity or low protein abundance may not be detectable in plasma. For instance, RSAD2 and GBP1 may exhibit high intracellular activity but are not secreted into the extracellular environment. The presence of CXCL10 and STAT1 in plasma reflects their role in extracellular signaling, while intracellular regulators like RSAD2 function upstream within cells. The consistent detection of STAT1, CXCL10, and OAS3 across both datasets highlights their critical roles in immune signaling, antiviral defense, and inflammation. The absence of genes such as RSAD2 and IFI44 in the plasma dataset suggests their functions are limited to intracellular processes. The GTEx dataset provides a broader perspective on gene activity across tissues, while the HPA dataset focuses on functional proteins in plasma, offering complementary insights into gene and protein roles [25,26]. Validating hub genes through these complementary databases strengthens the biological plausibility and relevance of our findings, particularly regarding their roles in immune response pathways and potential as biomarkers for influenza vaccination outcomes. In examining the correlation between TNFRSF17, JCHAIN, and antibody titers, the findings underscore the distinct roles these genes play in vaccine-induced immunity. TNFRSF17 is significantly associated with plasma cell activation (M4.11) and correlates with elevated antibody titers following vaccination. Conversely, although JCHAIN is highly expressed, it does not directly correlate with plasma cell overexpression; rather, it may be implicated in interferon-stimulated responses (M1.2, M3.4, M5.12). These results indicate that TNFRSF17 is directly involved in antibody production, whereas JCHAIN contributes to broader immune regulation. The data supporting these conclusions were obtained from GSE166545.

The study identifies differentially expressed genes (DEGs) and hub genes based on bioinformatics analysis, but no in vitro or in vivo validation was performed; hence, experimental studies such as qPCR, Western blot, and flow cytometry could confirm the biological relevance of key genes, including JCHAIN. Longitudinal studies with extended follow-up periods are required to assess the durability of immune responses beyond the first week after vaccination. Furthermore, integrating serological data, such as antibody titers and cytokine profiling, may reveal a direct link between transcriptomic changes and vaccine efficacy. Comparing participants with vaccinated non-pregnant women could help differentiate pregnancy-specific immune adaptations from overall vaccine responses. Furthermore, using advanced techniques like single-cell RNA sequencing may provide an improved comprehension of cellular and molecular interactions in response to vaccination. Expanding research to encompass diverse populations will strengthen the generalizability of outcomes, eventually contributing to the development of optimized, pregnancy-specific vaccine strategies to improve maternal and neonatal health outcomes.

## 4. Materials and Methods

### 4.1. Microarray Data and GEO Database

Maternal exposure to ultrafine particles enhances influenza infection during pregnancy, according to (https://www.ncbi.nlm.nih.gov/geo/query/acc.cgi?acc=GSE166545 (accessed on 15 October 2024)) [27], which serves as a publicly accessible repository for genomic data, encompassing high-throughput gene expression datasets from various experimental sources, including DNA microarrays. This investigation focuses on the analysis of microarray data from dataset GSE166545, which was retrieved using the search terms “Homo sapiens”, “Influenza, pregnant, AND vaccine”, and “Expression by array” [28,29]. The research employed the GPL10558 Illumina HumanHT-12 V4.0 Expression BeadChip, San Diego, California, USA, to characterize gene expression in whole blood samples obtained from pregnant women. At three different time points—Day 0 (pre-vaccination), Day 1, and Day 7 post-vaccination—132 samples were gathered. Following RNA extraction and globin reduction, labeled cRNA was hybridized to Illumina Human HT-12 BeadChips, all the data of this study in the Supplementary Materials [27].

### 4.2. Identification of DEGs

The identification of DEGs was accomplished using the GEO2R tool (https://www.ncbi.nlm.nih.gov/geo/geo2r/ (accessed on 4 December 2024)), an interactive web application developed by the National Center for Biotechnology Information (NCBI). The selection of time points for differential gene expression analysis (Days 0–1, Days 1–7, and Days 0–7) was based on the structure of the original study. The goal was to capture the following:

Early immune responses immediately after vaccination (Days 0–1 comparison); Later-stage immune responses to vaccination (Days 1–7 comparison); Overall gene expression changes spanning the full period from pre-vaccination to Day 7 (Days 0–7 comparison).

GEO2R utilizes the R packages limma and GEO query to facilitate comparisons between different sample groups. DEGs were identified based on a statistical significance threshold of adjusted *p*-value < 0.05 and |log2 fold change (FC)| ≥ 1 [28,29]. To determine biomarkers before and after vaccination, a more stringent filtering criterion was applied to the DEGs, using an adjusted *p*-value < 0.05 and |log2FC| ≥ 1 [29].

### 4.3. Construction of PPI Network

The construction of the PPI network was achieved using the STRING platform (http://string-db.org (accessed on 14 January 2025)), a comprehensive online resource that integrates known and predicted protein/protein association data for functional enrichment analysis. Visualization of the PPI network was performed using Cytoscape software (https://cytoscape.org (accessed on 14 January 2025, V3.10.3)) in conjunction with the STRING plugin, enabling an in-depth examination of the interactions within the dataset [28,29].

### 4.4. Identification of Hub Genes and Clusters

The cytoHubba plugin within Cytoscape was employed to identify hub genes within the PPI network (Cytoscape V3.10.3). These hub genes were ranked according to their connectivity and interaction significance using maximal clique centrality (MCC). The analysis incorporated a confidence score cutoff of 0.4. Cluster analysis was conducted using the Cytoscape MCODE plugin, with parameters set to a degree cutoff of 2, node density cutoff of 0.1, node score cutoff of 0.2, maximum depth of 100, and K-core of 2 [29].

### 4.5. Enrichment Analysis Using KEGG and Reactome Pathway for Hub Genes

To elucidate the biological functions and pathways associated with hub genes, KEGG and Reactome pathways analysis was performed using Enrichr (https://maayanlab.cloud/Enrichr/ (accessed on 14 January 2025)). These tools aggregate gene sets and biological data to provide qualitative insights into genomic sequences and their biological implications [29].

### 4.6. Validation of Hub Genes Utilizing Human Protein Atlas and GTEx Portal

To corroborate the top 12 hub genes identified through KEGG and Reactome pathway enrichment analyses, we employed two publicly accessible databases: the HPA and the GTEx Portal.

We extracted protein-level expression data for the hub genes from the HPA database (https://www.proteinatlas.org/ (accessed on 14 January 2025)). Our investigation centered on blood-related tissues, encompassing whole blood, lymphocytes, and additional immune cells. For each gene, we input its designation into the search interface, chose Homo sapiens, subsequently selected blood tissue, and exported the resulting data in graphical format. On the other hand, we acquired transcript-level expression data from the GTEx Portal (https://gtexportal.org/ (accessed on 14 January 2025)). The RNA expression levels of the hub genes were examined across whole blood and immune-associated tissues. We performed comparative analyses of transcriptomic profiles in GTEx to verify the presence and activity of these hub genes in blood and immune cells.

We compared the findings from the HPA and GTEx databases to assess the correspondence between protein and transcript-level expression for each hub gene. Genes demonstrating consistent expression patterns across both platforms were considered validated, lending support to their potential functional significance in immune responses and pathway activity.

To examine the association between TNFRSF17 and JCHAIN with antibody responses, transcriptional data from GSE166545 were analyzed. These genes exhibited the highest expression levels in blood samples, with JCHAIN showing significant activity in EBV-transformed lymphocytes, highlighting its role in immunoglobulin production and immune modulation. The correlation with antibody titers was assessed using Figure 9A,B, which depicts the relationship between gene expression levels and humoral immune responses following vaccination.

## 5. Conclusions

This study investigates the immunological response to influenza vaccination in pregnant women by analyzing differentially expressed genes (DEGs) and biological pathways at three specific time points: Day 0, Day 1, and Day 7 post-vaccination. The results indicated significant changes in gene expression, with 60 genes up-regulated and 12,854 down-regulated (Day 1 vs. 7), 55 genes up-regulated and 12,933 down-regulated (Day 0 vs. 1), and 2 genes up-regulated with no down-regulation (Day 0 vs. 7). Enrichment was noted in key pathways, including interferon alpha/beta (IFN-γ/β) signaling and toll-like receptor (TLR) signaling. Hub genes such as GBP1, RSAD2, CXCL10, and IFI44 were identified as the most significantly up-regulated (LogFc ≥ 1.5), playing crucial roles in the vaccine-induced immune response. Genes with moderate up-regulation (LogFc 1.0–1.5), such as STAT1 and OAS1, demonstrated notable activation. In contrast, JCHAIN exhibited minimal involvement (LogFc < 1.0) but was detected for the first time in pregnancy seven days post-vaccination. Validation results demonstrated that JCHAIN and TNFRSF17 exhibited the highest expression levels in blood samples. Subsequently, the correlation between these two genes and antibody titers was investigated. It was found that TNFRSF17 directly contributes to antibody production through plasma cell activation, whereas JCHAIN plays a broader role in immune regulation, potentially through interferon-mediated pathways. Validation of these hub genes confirmed their association with antiviral defense and vaccine efficacy. These findings elucidate the dynamic nature of immune responses in pregnant women following vaccination, identify potential biomarkers for vaccine efficacy, and underscore the need for tailored vaccination strategies to optimize maternal and neonatal health outcomes.

## Figures and Tables

**Table 1:** Genes exhibiting the highest up-regulation in differential expression analysis across three time intervals: Days (0–1), Days (1–7), and Days (0–7).

Gene Symbol	Gene Description	Log Fc	*p* Value
STAT1	Signal transducer and activator of transcription 1	1.033336	1.53 × 10^−18^
IFI35	Interferon-induced 35 kDa protein	1.189434	1.27 × 10^−13^
RSAD2	Radical S-adenosyl methionine domain-containing protein 2	1.639888	6.27 × 10^−8^
IFI44	Interferon-induced protein 44	1.636186	3.90 × 10^−9^
OAS1	2’-5’-oligoadenylate synthetase 1	1.026028	1.62 × 10^−8^
OAS3	2’-5’-oligoadenylate synthetase 3	1.395683	8.02 ×10^−10^
GBP1	Guanylate-binding protein 1	1.83377	6.80 × 10^−17^
IFIT3	Interferon-induced protein with tetratricopeptide repeats 3	1.043701	2.00 × 10^−8^
XAF1	XIAP-associated factor 1	1.141072	1.69 × 10^−11^
CXCL10	C-X-C motif chemokine 10	1.802314	4.18 × 10^−11^
TNFRSF17	Tumor necrosis factor receptor superfamily member 17	1.146	4.94
JCHAIN	Joining chain of multimeric IgA and IgM	0.967	5.482

## Data Availability

The data presented in this study are available upon request from the corresponding author.

## Supplementary Materials

The online version contains supplementary material available at https://www.ncbi.nlm.nih.gov/geo/query/acc.cgi?acc=GSE166545 (accessed on 15 October 2024).
